# Seed germination responses to seasonal temperature and drought stress are species‐specific but not related to seed size in a desert steppe: Implications for effect of climate change on community structure

**DOI:** 10.1002/ece3.4909

**Published:** 2019-01-25

**Authors:** Fengyan Yi, Zhaoren Wang, Carol C. Baskin, Jerry M. Baskin, Ruhan Ye, Hailian Sun, Yuanyuan Zhang, Xuehua Ye, Guofang Liu, Xuejun Yang, Zhenying Huang

**Affiliations:** ^1^ Inner Mongolia Research Center for Prataculture, State Key Laboratory of Vegetation and Environmental Change, Institute of Botany Chinese Academy of Sciences Beijing China; ^2^ Inner Mongolia Academy of Agricultural and Animal Husbandry Sciences Hohhot China; ^3^ University of Chinese Academy of Sciences Beijing China; ^4^ Department of Biology University of Kentucky Lexington Kentucky; ^5^ Department of Plant and Soil Sciences University of Kentucky Lexington Kentucky

**Keywords:** climate change, community structure, desert steppe, drought stress, seasonal temperature, seed germination, seed size

## Abstract

Investigating how seed germination of multiple species in an ecosystem responds to environmental conditions is crucial for understanding the mechanisms for community structure and biodiversity maintenance. However, knowledge of seed germination response of species to environmental conditions is still scarce at the community level. We hypothesized that responses of seed germination to environmental conditions differ among species at the community level, and that germination response is not correlated with seed size. To test this hypothesis, we determined the response of seed germination of 20 common species in the Siziwang Desert Steppe, China, to seasonal temperature regimes (representing April, May, June, and July) and drought stress (0, −0.003, −0.027, −0.155, and −0.87 MPa). Seed germination percentage increased with increasing temperature regime, but *Allium ramosum*, *Allium tenuissimum*, *Artemisia annua*, *Artemisia mongolica,* *Artemisia scoparia*, *Artemisia sieversiana*, *Bassia dasyphylla, Kochia prastrata,* and *Neopallasia pectinata* germinated to >60% in the lowest temperature regime (April). Germination decreased with increasing water stress, but *Allium ramosum*, *Artemisia annua*, *Artemisia scoparia*, *Bassia dasyphylla, Heteropappus altaicus*, *Kochia prastrata*, *Neopallasia pectinata,* and *Potentilla tanacetifolia *germinated to near 60% at −0.87 MPa. Among these eight species, germination of six was tolerant to both temperature and water stress. Mean germination percentage in the four temperature regimes and the five water potentials was not significantly correlated with seed mass or seed area, which were highly correlated. Our results suggest that the species‐specific germination responses to environmental conditions are important in structuring the desert steppe community and have implications for predicting community structure under climate change. Thus, the predicted warmer and dryer climate will favor germination of drought‐tolerant species, resulting in altered proportions of germinants of different species and subsequently change in community composition of the desert steppe.

## 1 INTRODUCTION

As the earliest life‐stage transition seed germination is a critical stage in the life history of plants because once seeds germinate seedlings either become established or die. Furthermore, seed germination may increase plant fitness if animals or pathogens attack seeds more than seedlings (Wenny, 2000). Timing of seed germination determines the conditions for subsequent seedling establishment and plant growth and thus is a crucial component of fitness (Donohue, Casas, Burghardt, Kovach, & Willis, 2010). Consequently, germination should be triggered by environmental cues that indicate the onset of conditions favorable for seedling establishment and thereby provide the opportunity for subsequent adaptive divergence to reduce extinction risk (Donohue et al., 2010; Willis et al., 2014).

Although germination response to climatic factors has been investigated for alpine/subalpine species at population and community levels (Cao et al., 2018; Cavieres & Arroyo, 2000; Giménez‐Benavides, Escudero, & Pérez‐García, 2005; Liu et al., 2011, 2018; Shimono & Kudo, 2005; Wagner & Simons, 2009), knowledge of seed germination response of species in temperate desert regions is scarce at the community level. Knowledge of differential responses seed germination within and between species in a community is important in predicting community composition under future climate conditions.

Variation in germination between species in a community can determine community structure. Plants have evolved strategies involving both predictive germination and optimization of their fitness such that some seeds germinate in the current environment, while others remain dormant and thus hedge their bet against unpredictable conditions that are not favorable for seedling establishment (Cohen, 1966; Gremer, Kimball, & Venable, 2016). In the desert, germination in different environments with distinct water and temperature conditions is a risk‐spreading strategy (Gremer & Venable, 2014; Snyder, 2006; Venable, Flores‐Martinez, Muller‐Landau, Barron‐Gafford, & Becerra, 2008; Volis & Bohrer, 2013). Furthermore, variation in germination requirements of desert plants at the community level may allow species coexistence, since it can contribute to temporal differences in use of resources such as water and nutrients between species and provide a buffer against extinction in the unpredictable desert environment (Ellner, 1987; Gremer & Venable, 2014). In addition, the differences between species in conditions required for germination can result in different combinations of annual species at the same site in different years (Chesson & Huntly, 1988; Venable, 2007).

Seed germination is sensitive to environmental conditions. Seed germination can occur only in response to specific combinations of environmental cues present in the field such as temperature regime, precipitation, or light (Donohue et al., 2010), although after dormancy is fully broken, seeds of many species can germinate over a wide range of conditions. Temperature requirements for dormancy break and germination have been the focus of much seed ecology research (Baskin & Baskin, 2014; Finch‐Savage & Leubner‐Metzger, 2006).

Seasonally fluctuating temperatures are an important factor determining germination time of nondormant seeds, and species in different sites show different germination behaviors in response to temperature fluctuation (Liu et al., 2013). For instance, species distributed at high elevations (>2,000 m) on the Tibetan Plateau did not show a significant germination response to temperature fluctuation, whereas those distributed over a broad elevation (both high and low) had a significant positive response (Liu et al., 2013). Thus, studying how fluctuating temperature (seasonally or diurnal) affects seed germination of species in different ecosystems can provide valuable information for understanding community structure and biodiversity maintenance.

Seed germination is also related to seed traits. One of the most studied regeneration traits is seed mass, which varies considerably among and within species, reflecting the many evolutionary forces acting upon this trait (Leishman, Wright, Moles, Westoby, & Fenner, 2000; Moles et al., 2005). At the global scale, environments with stable climate are expected to select for and be dominated by taxa that produce relatively large, fast‐germinating seeds, while relatively small seeds should be adaptive to and predominate in seasonal habitats (Rubio de Casas et al., 2017). Species with small seeds have high temporal variation in germination that spreads the risk of extinction, and large seeds produce large seedlings that perform better than small ones under stressful conditions (Kitajima & Fenner, 2000; Pake & Venable, 1996). Seedlings from large seeds usually can benefit from early emergence by maximizing development of deep root systems and thus defend against aboveground herbivory and climatic stress before the onset of the unfavorable season (Brunner, Herzog, Dawes, Arend, & Sperisen, 2015). In the Amazon region, tree species with small seeds commonly occur in transitional or seasonal forests, and large seeds are generally associated with climatically stable rainforests with high temperatures and precipitation (Malhado et al., 2015).

Large‐seeded species are expected to have higher post‐dispersal predation than small‐seeded species (Blate, Peart, & Leighton, 1998; Janzen, 1971) and thus should germinate promptly to avoid risks of mortality. Conversely, small‐seeded species are expected to delay germination and thus be persistent in the soil seed bank (Rees, 1994; Venable & Brown, 1988). Empirical data support this prediction for grasslands (Grime, Mason, Curtis, Rodman, & Band, 1981), but no conclusive evidence for it has been found in other temperate environments (Moles & Westoby, 2010; Leishman & Westoby, 1994).

Due to low rainfall and high evaporation, low water potential caused by drought stress also affects seed germination in deserts (Alvarado & Bradford, 2002; Volis & Bohrer, 2013). However, species vary considerably in response of seed germination to drought stress (Kos & Poschlod, 2008). Large seeds may buffer seedlings from the negative effects of drought (Leishman et al., 2000), and there is experimental evidence for the advantage of large seed size in establishment of plants under low soil moisture conditions (Leishman & Westoby, 1994). Therefore, seed size is expected to be positively correlated with the ability to germinate under drought stress. Germination only at high water potentials (low drought stress) is predictive of the environment for seedling growth since high water potential indicates a high rainfall event, which ensures that seeds germination and seedlings become established. Thus, low drought tolerance during seed germination (a cautious germination strategy) provides a fitness variance reducing mechanism and is expected to show negative correlations with other variance reducing life‐history attributes such as large seed size (Brown & Venable, 1986; Kos & Poschlod, 2008). Therefore, knowledge of relationships between germination response to drought stress and seed size is important for understanding the adaptive strategy of plants to desert environments.

Variation in seed germination of a species under different environmental conditions can increase long‐term reproduction of plant populations by temporally spreading risk and thus maximizing their fitness (Philippi & Seger, 1989; Simons, 2011; Venable, 2007). In deserts, seed germination of many species can only occur under a specific combination of temperature, light, and soil moisture (Adondakis & Venable, 2004; Finch‐Savage & Leubner‐Metzger, 2006). Germination timing is important in determining both survival probability and competitive success (Baskin & Baskin, 1972; González‐Astorga & Núñez‐Farfán, 2000; Venable, Dyreson, & Morales, 1995). If species in a plant community respond differently to environmental germination cues and seedlings survive, species‐specific responses would be a powerful contributor to differentiation of germination niche between species (Schwienbacher, Navarro‐Cano, Neuner, & Erschbamer, 2012).

With the aim of understanding seed germination strategies at the multi‐species scale in the desert environment, we tested the germination response of 20 common species with no or shallow dormancy in a desert steppe to seasonal temperature regimes and drought stress. We hypothesized that responses of seed germination to seasonal temperature and drought stress differ among species at the community level and that germination response is not correlated with seed size due to the adaptation of all species in the stressful environment. Specifically, we asked following questions: (a) How does seasonal temperature affect germination? (b) How does germination respond to drought stress? (c) Is germination related to seed traits?

## 2 MATERIALS AND METHODS

### 2.1 Seed collection

Fresh mature seeds of 20 species were collected in the Siziwang Desert Steppe from August to October in 2016 (see Table 1 for species list). The Steppe is located in Inner Mongolia Autonomous Region, China (41°47'17" N, 111°53'46" E; 1,450 m a.s.l.). Vegetation in the study area is dominated by *Artemisia frigida*, *Cleistogenes songorica*, *Convolvulus ammannii,* and *Stipa breviflora*. According to our field survey, there were 38 species in the steppe we studied. The community was mostly homogenous across the steppe. To study seed germination at the community level, we selected 20 common species in this region. These 20 species accounted for about 53% of the species in the community. Based on our preliminary study, we selected the 20 species with no or shallow seed dormancy that germinated to a relatively high percentage, making their germination responses to environmental variation comparable. For each species, seeds were collected at the beginning of their dispersal period from 10 individual plants that were at least 10 m from each other. In the laboratory, the seeds were cleaned by hand and air‐dried for 5 days before they were stored at −20°C for 1–7 months.

**Table 1:** Information on life cycle, growth form and seed mass and size of the 20 species included in the study

Species	Code	Life cycle	Growth form	1,000‐seed mass (g)	Seed length (mm)	Seed width (mm)
*Lagochilus ilicifolium*	LAIL	Perennial	Forb	6.384 (0.101)	4.48 (0.055)	2.86 (0.048)
*Allium ramosum*	ALRA	Perennial	Forb	3.467 (0.028)	3.66 (0.037)	2.65 (0.06)
*Allium polyrhizum*	ALPO	Perennial	Forb	1.876 (0.011)	2.50 (0.068)	1.67 (0.035)
*Linum stelleroides*	LIST	Annual	Forb	1.697 (0.057)	3.94 (0.037)	2.34 (0.031)
*Stipa breviflora*	STBR	Perennial	Grass	1.631 (0.017)	7.69 (0.178)	0.82 (0.02)
*Allium tenuissimum*	ALTE	Perennial	Forb	1.584 (0.018)	2.22 (0.033)	1.84 (0.034)
*Haplophyllum dauricum*	HADA	Perennial	Forb	1.139 (0.009)	2.35 (0.022)	1.46 (0.031)
*Kochia prastrata*	KOPR	Perennial	Shrub	0.88 (0.012)	1.77 (0.037)	1.33 (0.026)
*Bassia dasyphylla*	BADA	Annual	Forb	0.56 (0.05)	1.71 (0.048)	1.21 (0.028)
*Amaranthus retroflexus*	AMRE	Annual	Forb	0.438 (0.003)	1.37 (0.021)	1.27 (0.021)
*Heteropappus altaicus*	HEAL	Perennial	Forb	0.361 (0.007)	2.38 (0.044)	1.35 (0.022)
*Potentilla tanacetifolia*	POTA	Perennial	Forb	0.358 (0.001)	1.48 (0.039)	1.16 (0.016)
*Plantago depressa*	PLDE	Perennial	Forb	0.329 (0.005)	1.61 (0.026)	0.81 (0.013)
*Artemisia sieversiana*	ARSI	Annual	Forb	0.312 (0.002)	1.9 (0.054)	0.89 (0.029)
*Neopallasia pectinata*	NEPE	Annual	Forb	0.27 (0.005)	1.83 (0.015)	1.03 (0.021)
*Potentilla multicaulis*	POMU	Perennial	Forb	0.231 (0.006)	1.239 (0.019)	0.89 (0.025)
*Artemisia frigida*	ARFR	Perennial	Forb	0.106 (0.002)	1.3 (0.039)	0.57 (0.015)
*Artemisia mongolica*	ARMO	Perennial	Forb	0.098 (0.002)	1.66 (0.031)	0.56 (0.031)
*Artemisia annua*	ARAN	Annual	Forb	0.054 (0.001)	0.86 (0.016)	0.57 (0.015)
*Artemisia scoparia*	ARSC	Perennial	Forb	0.047 (0.002)	1.01 (0.06)	0.53 (0.034)

Note

Numbers in the parentheses are *SE*s. Annual species are all summer annuals. Species are organized by seed mass.

Temperature and rainfall data in the Siziwang Banner from 1981 to 2010 were obtained from the website of China Mereorological Administration, China Meteorological Data Service Center (http://www.cma.gov.cn). The study area has a typical temperate continental climate. Mean annual temperature is 3.4°C, and mean monthly temperature for April, May, June, and July is 6, 13, 18, and 21°C, respectively. Mean annual precipitation is 280 mm, with >73% of it occurring from June to September.

### 2.2 Measurement of seed size

The mass of 1,000 seeds was gravimetrically determined by weighing 10 replicates of 200 large seeds and of 500 small seeds using an electronic balance (1/10,000, Mettler Toledo, Switzerland). Seed size was determined by measuring the length and width of 10 randomly chosen seeds using vernier calipers (Xifeng, China). Seed area was calculated based on seed length and width using the ellipse equation.

### 2.3 Effect of temperature regime on seed germination

From meteorological data, we calculated mean monthly minimum and maximum temperatures in the study region. Four temperature regimes representing April, May, June, and July were used: 0 /12°C, 7/19°C, 12/24°C, and 15/27°C, respectively. The temperature regimes for April and May also approximated those in autumn in the steppe. At each temperature regime, seeds were exposed to 12 hr of dark and 12 hr of cool white fluorescent light (about 100 μmol m^−2^ s^−1^ of photosynthetically active radiation) each day. For each temperature regime and species, four Petri dishes of 25 seeds each were used as replicates. Seeds were placed in Petri dishes (9‐cm‐diameter) on two layers of filter paper moistened with 5 ml distilled water, after which they were sealed with Parafilm to minimize evaporation of water. Germination was monitored daily for 30 days. The criterion for germination was radicle protrusion >1 mm. Ungerminated seeds were prodded with forceps to determine whether the embryo was firm, indicating a viable seed (Baskin & Baskin, 2014).

### 2.4 Effect of drought stress on seed germination

To test the effect of drought stress on seed germination of each species, polyethylene glycol (PEG) was used to generate osmotic stress (Money, 1989). PEG 6000 (analytical grade) solutions were used in concentrations of 0 (distilled water control), 2.5, 5, 10, and 20% (w/v). Osmotic potentials, calculated using the formula from Money (1989) for PEG 6000, were 0.0, −0.003, −0.027, −0.155, and −0.87 MPa, respectively. For each water potential, four replicates of 25 seeds per species were used. Seeds were placed in a Petri dish (9‐cm‐diameter) on two layers of filter paper moistened with the appropriate solution and sealed with Parafilm to minimize evaporation. Seeds were incubated in 12 hr of dark and 12 hr of light (as above) at 25°C, and germination was monitored daily for 30 days.

### 2.5 Statistical analysis

Germination data were arcsine transformed to ensure homogeneity of variance before performing an ANOVA. Germination data were analyzed by two‐way ANOVA, in which species and temperature regime or PEG concentration were treated as fixed factors. To explore the relationships between mean germination percentages in different conditions (temperature regime and drought stress) and seed traits, standardized major axis regressions were performed. Germination percentages were arcsine‐transformed and seed mass/area log‐transformed before analyses. R package “smatr” (Warton, Duursma, Falster, & Taskinen, 2012) was used for standardized major axis regressions.

To determine the effects of phylogeny on seed germination response, we generated a phylogeny for our species list using Phylomatic (Webb & Donoghue, 2005). We scaled branch lengths using known node ages from Wikstrom, Savolainen, and Chase (2001) with the BLADJ procedure in PHYLOCOM 4.2 (Webb, Ackerly, & Kembel, 2008). The phylogenetic distance estimates are in millions of years. We used the phylosig function of the phytools package to calculate Blomberg's *K*, which measures the phylogenetic signal or the tendency of related species to resemble each other (Blomberg, Garland, & Ives, 2003). All data were analyzed in R 3.4.1 (http://www.R-project.org).

## 3 RESULTS

### 3.1 Species and seed size

The 20 study species (7 annuals and 13 perennials) included 18 forbs, one grass, and one shrub species (Table 1). There was a large difference (135‐fold) in 1,000‐seed mass between species (0.047 ± 0.002 g for *Artemisia scoparia* and 6.384 ± 0.101 g for *Lagochilus ilicifolium*). Seed length varied (8.9‐fold) from 0.86 ± 0.016 mm for *Artemisia annua* to 7.69 ± 0.178 mm for *Stipa breviflora*, and seed width (5.4‐fold) from 0.53 ± 0.034 mm for *Artemisia scoparia* to 2.86 ± 0.048 mm for *Lagochilus ilicifolium*.

### 3.2 Effect of temperature regime on seed germination

**Figure 1:**
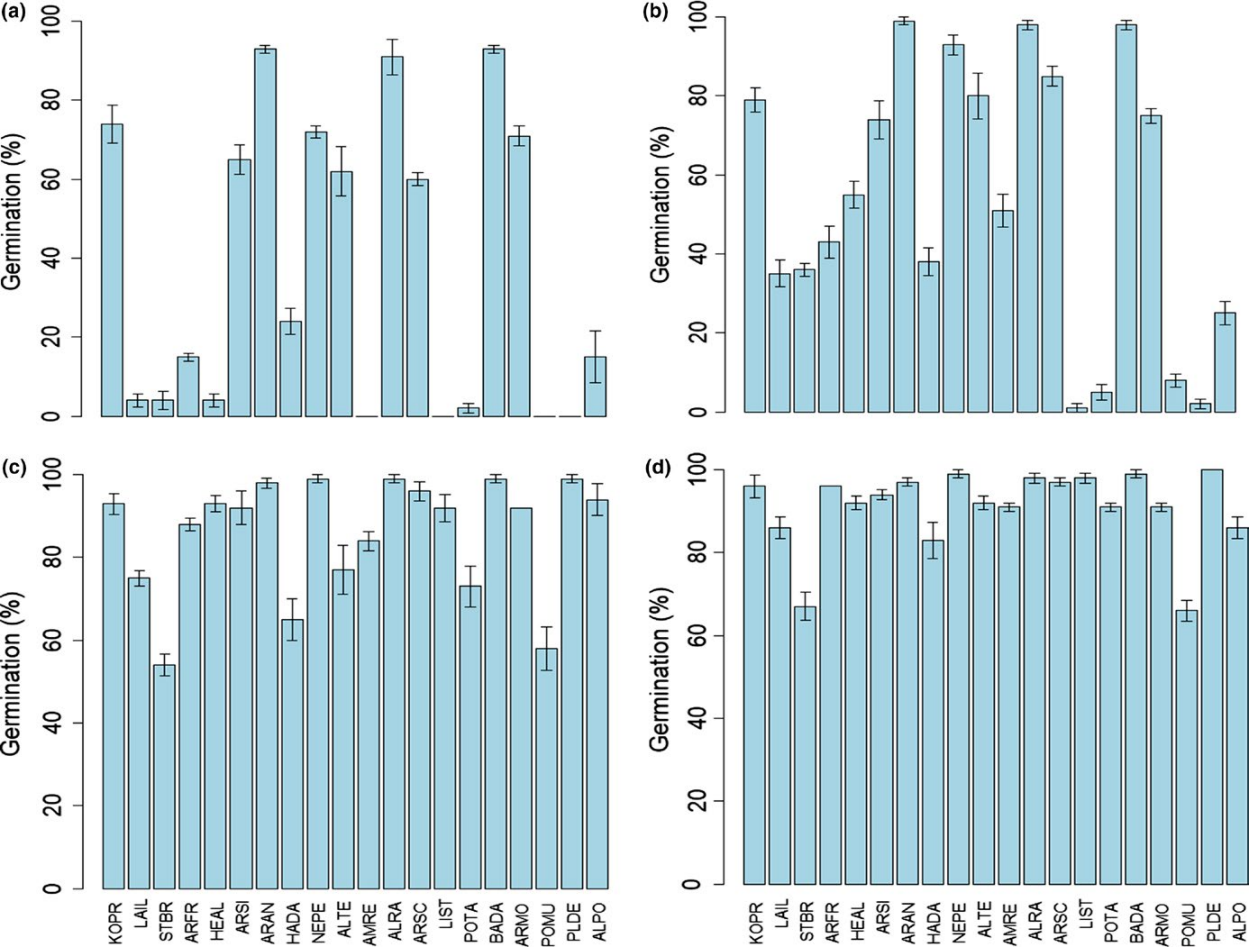
Seed germination of the 20 species at (a) April (0 /12°C), (b) May (7/19°C), (c) June (12/24°C), and (d) July (15/27°C) temperature regimes. Bars represent ±1 *SE*. See Table 1 for species code

Germination differed significantly among species (*F* = 131.27, *p* < 0.001) and temperature regimes (*F* = 945.5, *p* < 0.001). Germination percentage increased with increased temperature (Figure 1). At the lowest temperature regime (April), seeds of *Allium tenuissimum*, *Allium ramosum*, *Artemisia annua*, *Artemisia mongolica, Artemisia scoparia*, *Artemisia sieversiana*, *Bassia dasyphylla,* *Kochia prastrata,* and *Neopallasia pectinata* germinated to >60% (Figure 1a); at May temperatures germination of six other species increased to near 40% (Figure 1b); at June temperatures germination percentages of all species increased (Figure 1c); and at the highest temperature regime (July) all species germinated to the highest percentage (mostly >80%; Figure 1d).

### 3.3 Effect of drought stress on seed germination

**Figure 2:**
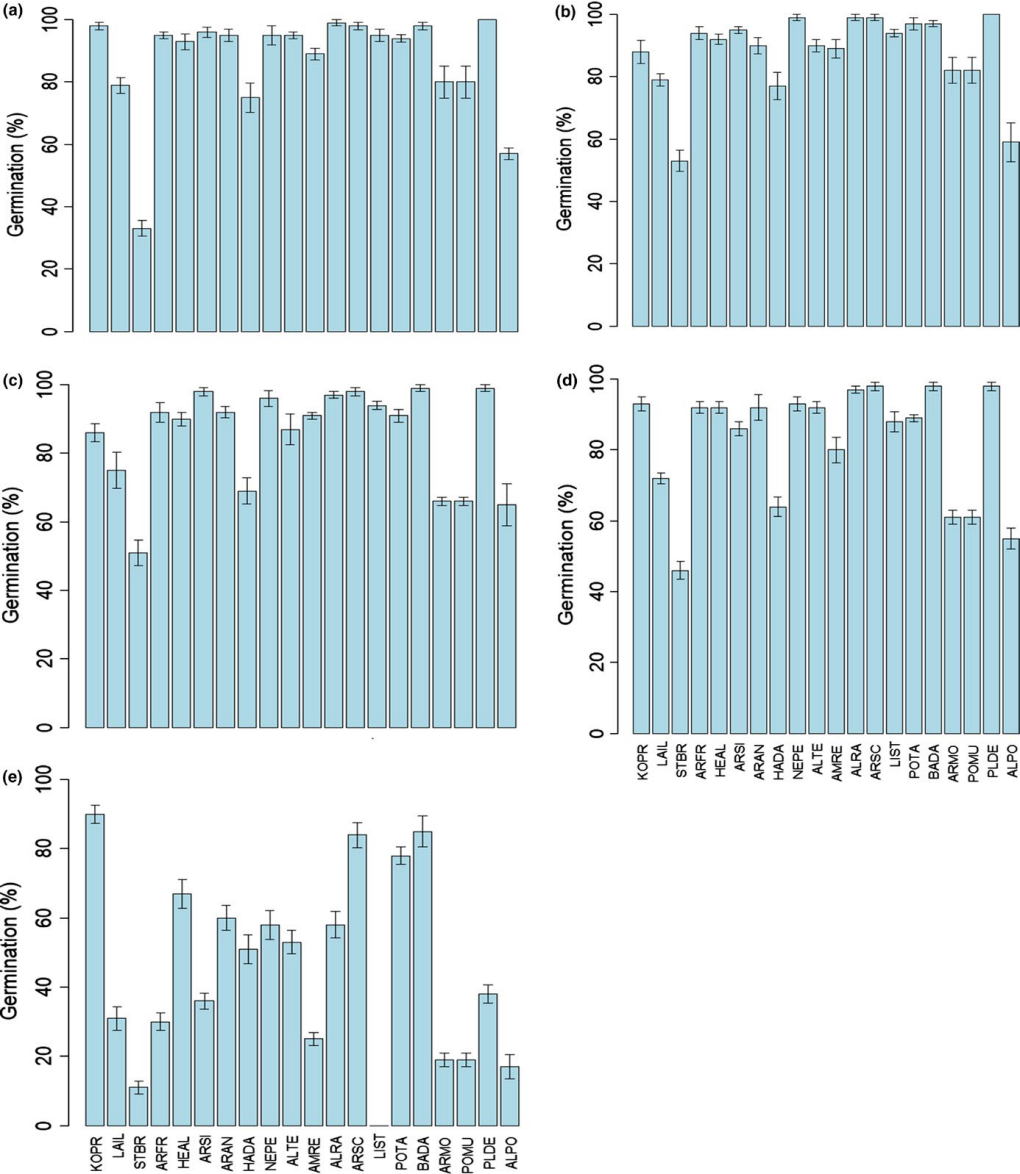
Germination of seeds of the 20 species incubated in light at 25°C at water potentials of (a) 0, (b) −0.003, (c) −0.027, (d) −0.155, and (e) −0.87 MPa. Bars represent ±1 *SE*. See Table 1 for species code

**Figure 3:**
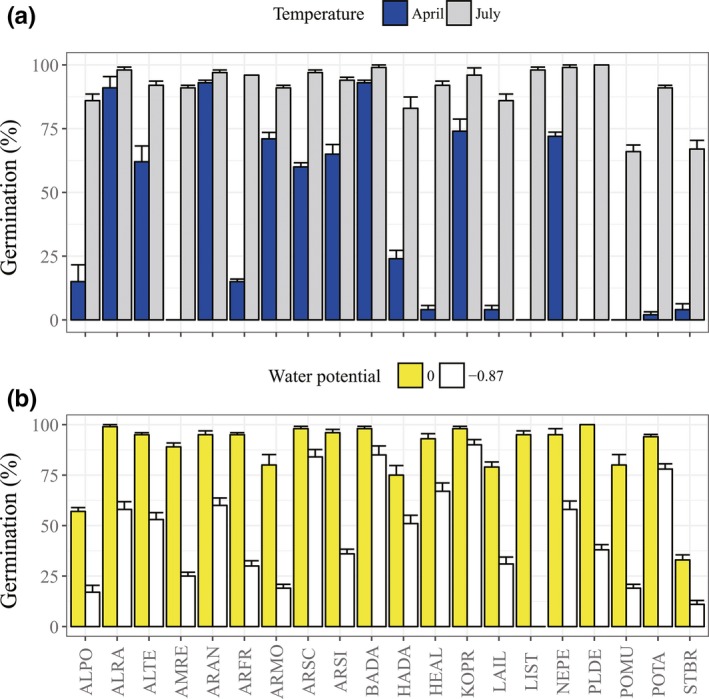
Seed germination of the 20 species at the lowest and highest temperature regimes (a) and water potentials (b). Bars represent ±1 *SE*. See Table 1 for species codes

Germination percentage differed significantly among species (*F* = 53.43, *p* < 0.001) and water potential (*F* = 906.55, *p* < 0.001). For all species tested, germination percentage decreased with increased water stress (Figure 2). Germination percentage of most species was not affected when water potential was higher than −0.155 MPa (Figure 2a–d). However, at the highest drought stress (−0.87 MPa) germination percentage of most species decreased, but seeds of *Allium ramosum*, *Artemisia annua*, *Artemisia scoparia*, *Bassia dasyphylla,* *Heteropappus altaicus*, *Kochia prastrata*, *Neopallasia pectinata, and* *Potentilla tanacetifolia* germinated to near 60% (Figures 2e and 3).

### 3.4 Relationships between seed germination and seed traits

**Figure 4:**
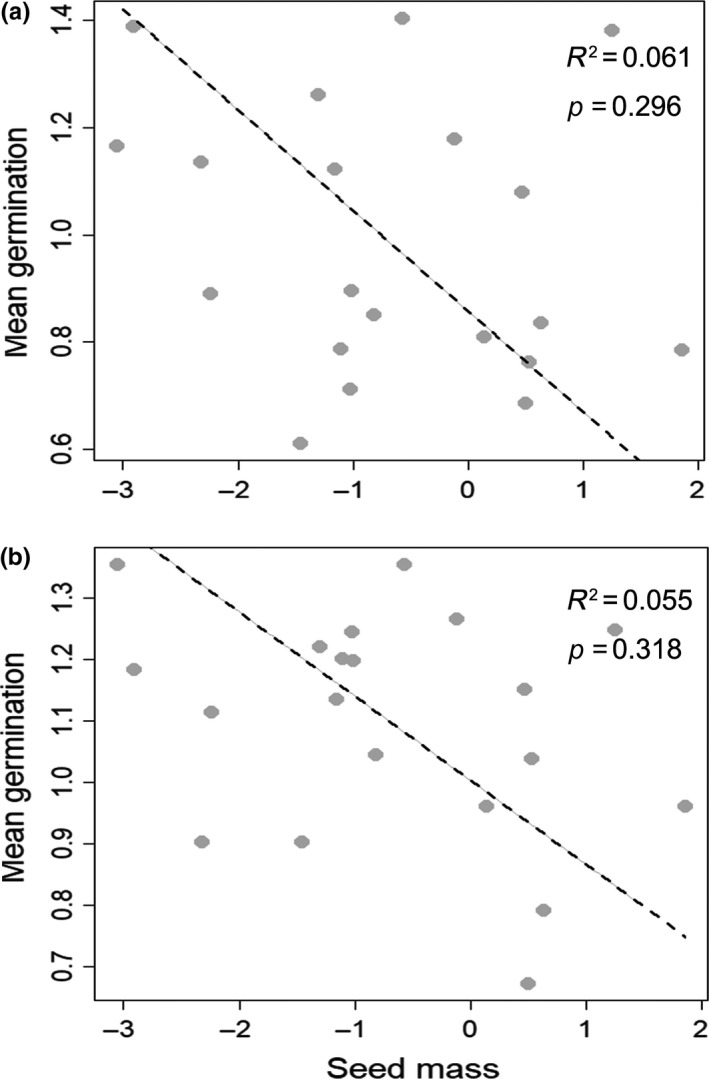
Relationships between mean seed germination percentage and seed size. (a) Temperature regime and seed mass; (b) Water potential and seed mass. Mean germination percentage was arcsine‐transformed and seed mass/area log‐transformed

Mean germination percentage in the four temperature regimes and the five water potentials was negatively correlated with seed mass, but these relationships were not significant (Figure 4a,b). Similarly, mean germination percentage was negatively correlated with seed area, which also was not significant (for temperature: *R* ^2^ = 0.055, *p* = 0.318; for water potential: *R* ^2^ = 0.103, *p* = 0.167).

Blomberg's *K*‐values for mean germination at the four temperature regimes and the five water potentials were 0.166 (*p* = 0.076) and 0.11 (*p* = 0.416), respectively. This indicates that phylogeny does not play an important role in determining seed germination in the desert steppe community.

## 4 DISCUSSION

In our study, species responded differently to seasonal temperature regimes, especially to those of April and May (Figure 1), indicating that they might differ in time of seedling establishment in the desert steppe. A study in the Sonoran (hot) Desert reported species‐specific germination timing in which some species readily germinate in the early fall, while others preferentially germinate in winter (Venable, 1989). Although seeds of all our study species had no or shallow dormancy, they are unlikely to germinate immediately after seed dispersal (in autumn). If seeds are nondormant, they may not be able to germinate because it is too dry in the habitat in autumn. If seeds have shallow dormancy, then they undergo dormancy break in winter and/or early spring and germinate in spring or summer. Our results show that seeds of* Allium ramosum*, *Allium tenuissimum*, *Artemisia annua*, *Artemisia mongolica, Artemisia sieversiana, Artemisia scoparia*, *Bassia dasyphylla*, *Kochia prastrata,* and *Neopallasia pectinata *have higher germination under low temperature regimes than other species (Figure 1a), suggesting they can emerge in early spring. However, this prediction needs to be viewed with caution because the low germination of some species at low temperatures might be due to physiological dormancy that would have been broken by cold stratification during winter, resulting in the ability of seeds to germinate at low spring temperatures (Baskin & Baskin, 2014). Therefore, field seedling emergence in spring may be different from the prediction based on germination responses of species to low temperatures in our study. Nevertheless, our comparison between multiple species at different temperature regimes reveals that species‐specific responses to seasonal temperatures (different responses even in the highest temperature) could be important for the differentiation of germination niche between species in the temperate desert steppe.

In addition to differential germination responses to temperature, we found a heterogeneous set of germination responses to drought stress among the 20 study species. Responses to temperature and drought stress suggest a complex set of germination strategies. Therefore, seedlings of species that are relatively drought resistant are expected to germinate over a wider range of water potentials than those with low drought resistance (Kos & Poschlod, 2008). In our study, seeds of *Allium ramosum*, *Artemisia annua*, *Artemisia scoparia*, *Bassia dasyphylla,* *Heteropappus altaicus*, *Kochia prastrata*, *Neopallasia pectinata*, and *Potentilla tanacetifolia* germinated up to 60% even in −0.87 MPa (Figure 2), indicating that some species in the community have high tolerance to drought stress.

Rainfall in deserts and steppes can vary substantially among years in both amount and timing, and thus successful seedling establishment may be followed by complete reproductive failure (Beatley, 1967; Burk, 1982; Jakobsson & Eriksson, 2000; Rosbakh & Poschlod, 2015; Tevis, 1958). Therefore, germination under drought stress is a high‐risk strategy since the seedlings may die if the amount of follow‐up precipitation is not enough to support seedling growth. At the community level, this risk could be spread by heterogeneous germination under drought stress among species.

Our results are consistent with the empirical data of winter annuals in Sonoran Desert showing that coexisting species differ in their germination response to varying temperature and precipitation (Adondakis & Venable, 2004). Therefore, the difference in the responses of germination to water stress between species in our desert steppe community spreads germination timing, which could mitigate the crowding effect in the resource‐limited (e.g., water) ecosystem. Furthermore, species partition their regeneration niche through different responses to seasonal temperature regime and drought stress (Chesson & Huntly, 1988; Pake & Venable, 1996).

We found great variation in the response of seed germination to environmental conditions both within and between species. As an example of within‐species response, seed germination of *Allium polyrhizum* responded to temperature but remained constant as water potential changed. At the between species response level, seed germination of *Lagochilus ilicifolium* was extremely sensitive to temperature, but within the same community *Bassia dasyphylla* had little to no response. This variation would allow germination of different species as well as individuals of the same species to differ in space and time in the desert steppe community. In addition, the variation in seed germination has implications for predicting community change under predicted climate change. For example, the predicted warmer and dryer climates would favor germination of *Artemisia scoparia*, *Bassia dasyphyl*, *Kochia prastrata,* and *Potentilla tanacetifolia* over that of other species in the community, which would result in altered proportions of germinants of different species and subsequently change community composition of the desert steppe.

Theoretical models predict that small seeds are more likely to delay germination than large seeds (Rees, 1994; Venable & Brown, 1988). Compared with small seeds, large‐seeded species have high fitness due to high seedling survival (Westoby, Falster, Moles, Vesk, & Wright, 2002), rapid seedling growth (Moles & Westoby, 2004; Verdú & Traveset, 2005), and an amplified tolerance against hazards such as drought and frost (Coomes & Grubb, 2003). Some studies have reported that germination and establishment success increase with increasing seed mass in a variety of environmental conditions (Jakobsson & Eriksson, 2000; Moles, Westoby, & Eriksson, 2006). In addition, large seeds with large reserves of stored food produce larger seedlings that can perform better under unfavorable conditions than seedlings from small seeds (Kitajima & Fenner, 2000). Our results showed that there was no significant relationship between seed germination and seed size. However, there is much variation with regard to whether large or small seeds germinate best (Baskin & Baskin, 2014).

Length of growing season might be the core factor that affects maximum attainable seed mass because a longer growing season provides more time for nutrient accumulation than a shorter growing season (Moles & Westoby, 2010). Thus, selection will thus be more likely to favor large seeds in wet tropical environments, where a high‐energy period extends for all or most of the year. However, for species in the desert there is limited time to complete their lifespan and to produce large seeds (Volis & Bohrer, 2013). The nonsignificant relationship between seed size and mean germination percentage at different temperature/water stress conditions in our study suggests that regardless of their seed size most species in the desert steppe have the ability to germinate under low temperature and drought stress in the early season, which would increase plant fitness by increasing growing time. Our results do not support the general assumption that large‐seeded species necessarily germinate to higher percentages than small‐seeded species (Easton & Kleindorfer, 2008; Grime et al., 1981; Norden et al., 2009; Rees, 1994; Venable & Brown, 1988). However, our results are consistent with those of a study of 1795 species at the global scale reporting that the distributions of seed mass of species with dormant and nondormant seeds strongly overlaps (Jurado & Flores, 2005). In addition, our study also is consistent with a study of foreland species in the Austrian Central Alps showing no correlation between the ranking of species according to seed mass and optimum temperature for germination (Schwienbacher et al., 2012).

In addition, seed size is expected to be positively correlated with the ability to germinate under osmotic stress. Most research has concluded that large seeds buffer seedling growth from some of the negative effects of drought stress (Leishman et al., 2000), and experimental evidence for the advantage of large seeds for establishment under low soil moisture conditions has been reported (Leishman & Westoby, 1994). However, the relationships between germination under drought stress and seed size might differ among ecosystems. Therefore, determining the relationship between seed germination and seed size is important for predicting future community structures from this important seed trait (i.e., seed size). In our study, seeds of all species originated from the same temperate arid habitat with only 280 mm annual precipitation, in which most species are exposed to drought stress. Our findings of nonsignificant relationships between seed germination under drought stress and seed size are consistent with results for communities in other temperature regions such as a woodland in Massachusetts (USA) showing that seed size does not have a significant effect on emergence time of *Impatiens capensis* (Howell, 1981), forest understories in South Carolina (USA) showing that seedling emergence is not related to seed size (Jones, Allen, & Sharitz, 1997), and a glacier foreland in the Austrian Central Alps showing that seed mass does not correspond with germination along a temperature gradient (Schwienbacher et al., 2012). These studies, together with ours, suggest that seed germination in the temperate region may be more related to local environmental conditions or genetic heterogeneity than to seed size (Jones et al., 1997; Schwienbacher et al., 2012).

In summary, our study has shown how seed germination of species in the desert steppe community responds to two key environmental factors, that is seasonal temperature and drought stress. Our results suggest that most species are well‐adapted to the desert by the ability to germinate in a habitat characterized by cold and dry conditions. However, they are able to partition their regeneration niche through a heterogeneous set of germination responses to low temperature and drought stress in the early season. Furthermore, seed germination in our desert steppe community is not significantly related to seed size, which may be due to (i) the adaptation of seed germination to the desert conditions regardless of seed size, and/or (ii) the same environmental conditions that seeds experienced during their development. Therefore, our study points to the importance of environmental conditions in shaping seed germination response to desert environments at the community level.

## CONFLICT OF INTEREST

The authors declare that they have no conflict of interest.

## AUTHOR CONTRIBUTIONS

XJY and ZH conceived the ideas and designed the methodology; FY, YZ, RY, HS, XHY, and GL collected the data; XJY and ZW analyzed the data; FY, ZW, CCB, JMB, XJY, and ZW led the writing of the manuscript.

## Data Availability

Relevant data will be available via Dryad.
